# The Effect of Creators’ Personality Traits and Depression on Teamwork-Based Design Performance

**DOI:** 10.3390/bs13030248

**Published:** 2023-03-11

**Authors:** Szu-Jung Wang, Rain Chen, Hsiu-Ching Lu

**Affiliations:** 1 Toast International Co., Ltd., Taichung City 407, Taiwan; shirong0912@gmail.com; 2 Department of Visual Communication Design, Southern Taiwan University of Science and Technology, Tainan City 710, Taiwan; 3 Department of Styling and Cosmetology, Tainan University of Technology, Tainan City 710, Taiwan; t80058@mail.tut.edu.tw

**Keywords:** personality traits, depression, teamwork, design performance

## Abstract

Many organizations encourage carrying out a project by teamwork for consensus building and managing risks in decision making. When working as a team, the members’ personality traits and depression may affect project performance. This study explored the personality traits and depression of creators on their design performance in teamwork. This study used the Big-Five Personality Traits Scale (Big-5) and the Center for Epidemiologic Studies Depression Scale (CES-D). Forty-four volunteers were chosen to participate in this study as the study subjects. A total of 11 design teams were formed based on the participants’ personality trait score and the depression score. The design performance of the participants was rated by creativity, aesthetics, and completeness. The study results showed that (1) for creativity, PT4 Neuroticism and D1 No Depression performed the best; (2) for aesthetics, PT4 Neuroticism, PT5 Openness, and D2 Mild Depression performed the best; (3) for completeness, PT5 Openness and D4 Severe Depression performed the best; and (4) for member satisfaction and work satisfaction, PT4 Neuroticism and D2 Mild Depression had the lowest scores. Therefore, the overall teamwork performance can be affected by personality traits and depression. Each individual’s personality trait and emotional expression may improve team performance via different dimensions, such as creativity, aesthetics, and completeness. Project managers should have members of different personality traits and with different emotional expression on a team as their personality traits and emotion can facilitate team collaboration.

## 1. Introduction

To carry out a project, most organizations prefer the teamwork approach for building consensus and reducing risks in decision making. Teamwork is also preferred because it enhances team members’ interaction and creativity [1]. There are various factors influencing the performance of teamwork; some examples are personality traits, employee relationships, job stress, leadership, and management development [2]. Moreover, each team member has their unique personality and reasoning, which could affect team performance as well [3].

Personality traits are an individual’s explicit characteristics and temperament. Personality traits are also the distinct personality expressed by an individual when they interact with other people or adapt to the environment [4]. These characteristics are expressed by a person’s behavior and response [5].

When working as a team, people with the personality trait of agreeableness are willing to share their knowledge. People with the personality trait of neuroticism tend to have low self-esteem. They are also more emotional and have difficulty adapting [6]. As for people with the personality trait of extraversion, they like to communicate with others [7], and they are sociable and collaborative [8,9,10]. However, not all personality traits support creativity [11]. Previously, researchers exploring personality traits put more emphasis on job performance from the personal level, but in recent years, researchers from various fields have shifted their focus to the personality traits of members on a team.

Personality traits are permanent [12], and they predict an individual’s behavioral response in an environment. Emotion, on the other hand, occurs at the moment when an individual is aroused, and it is a process about a person relating and adapting to the environment [13]. People’s emotion is pluralistic. Aside from passion, anger, sadness, and happiness, there are many other more sophisticated emotions and emotions that are hard to distinguish. When people are in a stressful situation, they are affected both psychologically and physically, and stress will also be reflected on their emotion, which in turn may influence team performance [14,15,16,17,18,19,20].

In history, many well-known artists, composers, and writers, such as Vincent Willem van Gogh, Pyotr Ilyich Tchaikovsky, and Friedrich Wilhelm Nietzsche, had something in common—they all suffered from mental illness during their lifetime. Compared with ordinary people, visual artists and writers are more prone to mental health problems, and in certain fields, high creativity is associated with mental health problems. In fact, negative emotions may lead to higher creativity [21].

Among many negative emotions, depression is a mental health problem relatively prevalent among artists and designers. Lin (1991) once explored depression by comparing members of 86 depression families and non-depression families [22]. She found that depression families in comparison with non-depression families are fuzzier and more indirect in communication (e.g., passing a message or exchanging information). That is, when communicating with others, people with depression cannot precisely express their points as people without depression would.

Nevertheless, depression is not all negative. In the field of art and design, depression brings creativity. Nevertheless, depression may impede group communication. It is therefore worth exploring the effect of depressed team members on a team’s design performance and communication.

Taken together, related studies are focused on team members’ personality traits and performance [23,24,25]. Relevant studies also support the notion that creators are more sensitive than non-creators and may be depressive emotionally in the design process [26]. In this study, personality traits and depression are the independent variables, while design performance is the dependent variable. This study explores the differences in collaborative design by teams of designers with different personality traits and depression levels.

## 2. Materials and Methods

### 2.1. Subjects

Team members’ different family backgrounds and lifestyles affect team performance. Related studies proposed that the best number of people for teamwork is three to four people per team [27]. This study recruited 98 volunteers to participate in the experiment, and the Big-Five Personality Traits Scale (Big-5) and the Center for Epidemiologic Studies Depression Scale (CES-D) were implemented among the volunteers. Afterward, 44 qualified participants were selected and grouped for the experiment. The number of participants per team was 4, and therefore, a total of 11 teams (6 personality trait teams and 5 depression teams) were formed.

### 2.2. Samples

Story Cube, a board game for creativity and imagination, was designed by Rory O’Connor in 2005. This study picked six images of different dimensions for the image cards in the experiment [28]. Each image card was 70 mm × 70 mm. The four participants of each team had to collaborate to discuss the meaning of each of the six cards and to create a new graphic work.

### 2.3. Experimental Variables

Team-based design projects are a mainstream of practical design practice, but whether the collaboration is successful or not is affected by the personality traits and emotional characteristics of the designers. In fact, the substantive performance of a design team is often influenced by how designers carry out effective or creative communication. Related studies have shown that personality traits directly affect a design team’s performance. For example, extroverts tend to have better communication skills and are good at reducing conflict in the organization [5,7,8,9,10]. Aside from personality traits, designers are more prone to depression than others [29]. When designers with different levels of depression work collaboratively in design, their design performance will be affected by the levels of their depression [30]. These research findings suggest that personality traits and depression tendency may affect the general performance of the collaboration. Taking this one step further, this study examines differences in collaborative design by teams of designers with different personality traits and depression levels.

This study explored whether there is any difference in teamwork-based design performance when the team members have different personality traits and different levels of depression. The independent variable in this study was the personality trait team vs. the depression level team. The dependent variables were design performance and self-rated performance. A total of eleven independent design teams were formed.

For personality traits, there were six teams: PT1 Extraversion, PT2 Agreeableness, PT3 Conscientiousness, PT4 Neuroticism, PT5 Openness, and APT Averaging Personality Traits. For depression levels, there were five teams: D1 No Depression, D2 Mild Depression, D3 Moderate Depression, D4 Severe Depression, and AD Averaging Depression. For their design performance, the graphic work was rated by creativity, aesthetics, and completeness. In terms of self-rated performance, the indicators were member satisfaction and work satisfaction.

### 2.4. Experimental Design

First, the participants were asked to fill out Big-5 and CES-D. They were arranged in separate rooms based on their experimental group to eliminate interference from the external environment. Before starting the experiment, the researchers gave instructions on the experiment and informed the participants of the method, procedure, and rating criteria of the experiment.

Each team was given six experimental image cards and one sheet of blank drawing paper of B3 size. The participants used a black sign pen to create the image on the drawing paper based on their discussion. The time allowed for discussion and composition was limited to 15 min. Each team decided the amount of time used for planning, brainstorming, and drawing. Each team was asked to provide a description for or a story behind their work. Each participant was also asked to fill out the self-rated questionnaire.

### 2.5. Evaluation Instruments

#### 2.5.1. Big-5

Goldberg’s Big-Five Personality Traits Scale (Big-5) is a popular instrument used by researchers for the classification of personality traits. The five major personality trait types are extraversion, agreeableness, conscientiousness, neuroticism, and openness [31]. Several follow-up studies have been conducted using assorted data, samples, and assessment instruments, and they found an explanatory model similar to Goldberg’s five major personality traits [32]. Related studies also demonstrated that Big-5 has adequate reliability and validity [33,34,35,36]. Therefore, this study used Big-5 for personality trait grouping.

#### 2.5.2. CES-D

Depression is an emotional state or emotional disturbance. Some symptoms associated with depression are bad moods and sorrows [37]. To assess the level of depression in this study, the researchers used the Center for Epidemiologic Studies Depression Scale (CES-D). CES-D is a self-administered questionnaire that has been frequently used in multi-ethnic studies. It has a good reliability and validity [38]. There are 20 questions in CES-D. The score ranges from 0 to 60 points. The higher the score, the higher the level of depression. For adults, the cut-off score for depression is 16 points or greater [39]. For CES-D scoring, 0 to 15 points, subclinical depression; 16 to 20 points, mild depression; 21 to 30 points, moderate depression; more than 31 points, severe depression. This study used the above ranges for grouping the participants.

#### 2.5.3. Design Performance

The indicators of good design are multidimensional; some basic indicators are creativity, novelty, and practicability [40]. This study collected the rating criteria used in international design contests and finally came up with three design performance indicators for rating graphic design, and a weight was assigned to each of these indicators. The three indicators are creativity (30%), aesthetics (30%), and completeness (40%). This study invited five design experts to rate the design performance of the participants’ works. All experts had more than 5 years of design-related work experience, and three of them had worked in design for more than 20 years.

## 3. Results

### 3.1. Works and Comments

#### 3.1.1. PT1 Extraversion

**Figure 1:**
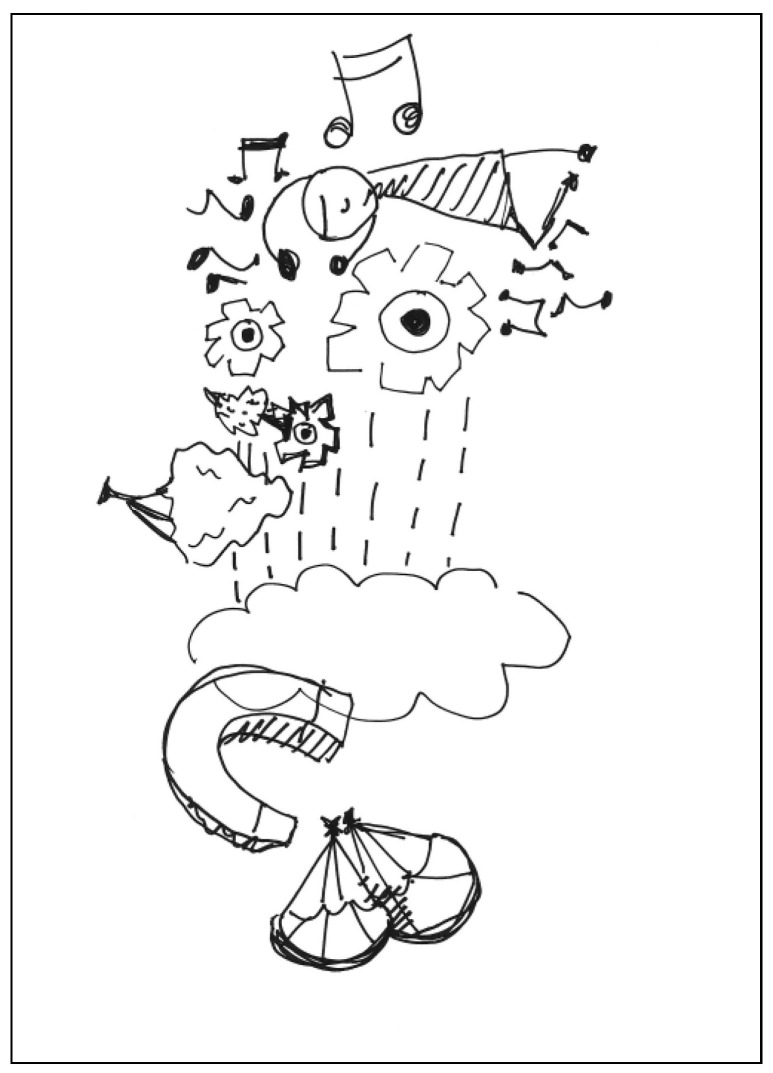
Work by PT1 Extraversion.

The description for PT1’s work: This is a tall tree that looks like the face of a man. The nose is like rain, the beard looks like clouds, the mouth is like a piece of magnet, and the chin is like a parachuter. See Figure 1. General comments from the experts: (a) The completeness is relatively low, (b) the lines and strokes are a little bit messy, (c) the elements are unconventional, and (d) the lines have a childlike playfulness.

#### 3.1.2. PT2 Agreeableness

**Figure 2:**
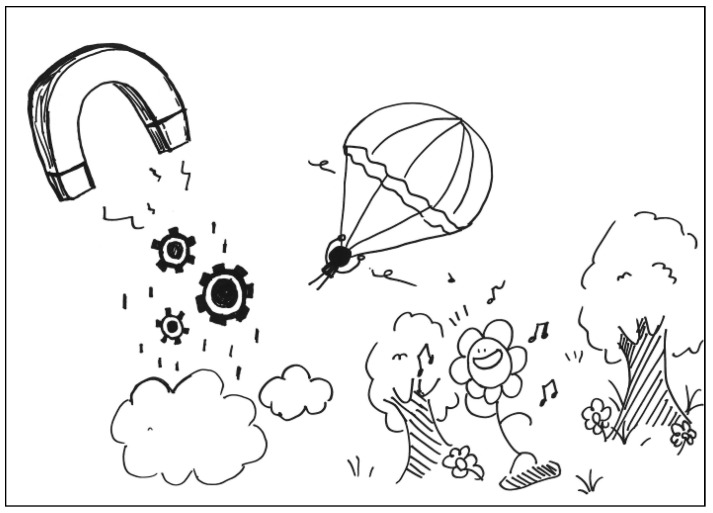
Work by PT2 Agreeableness.

The description for PT2’s work: The gears symbolize interactions between people. When a gear is attracted (controlled) by the magnet, it is like a bird in the cage losing its freedom. See Figure 2. General comments from the experts: (a) The structure of the story is too loose, (b) it has a poor sense of space, (c) the elements are poorly related, (d) there is a lack of visual tension, and (e) the elements are too concrete.

#### 3.1.3. PT3 Conscientiousness

**Figure 3:**
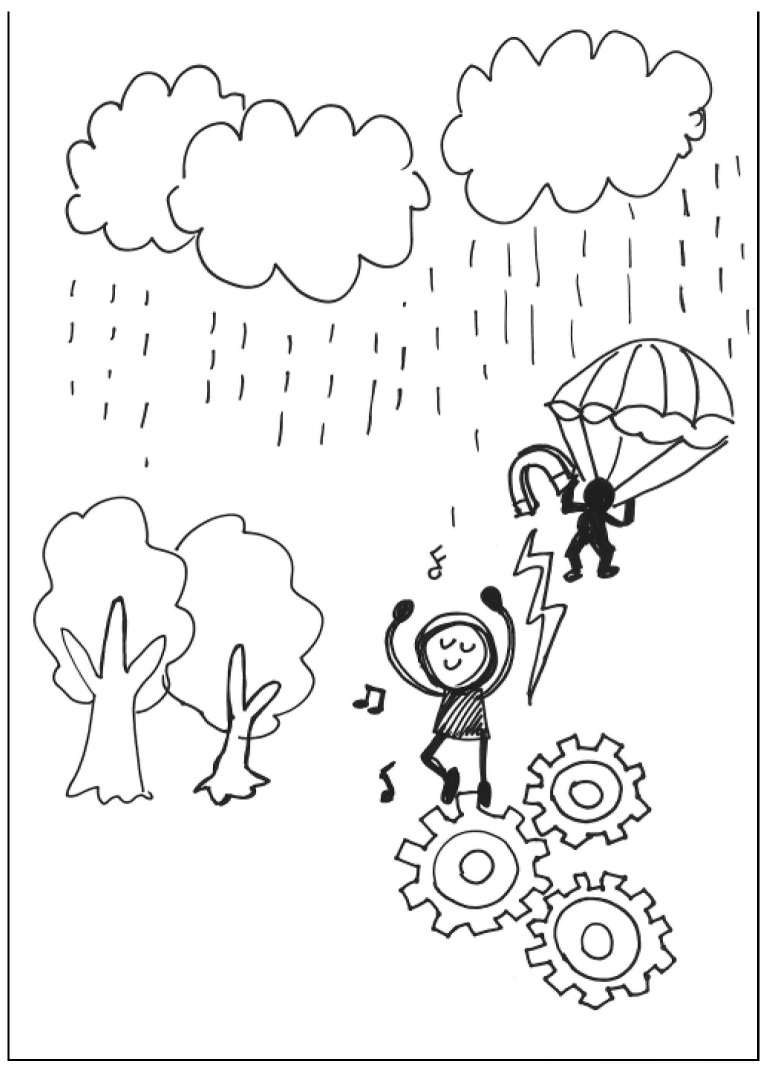
Work by PT3 Conscientiousness.

The description for PT3’s work: On rainy day in a forest, a man with a parachute gradually descends from the sky. He is holding a magnet to drive the gears on the ground. The gears in turn drive a man singing and dancing happily. See Figure 3. General comments from the experts: (a) The elements are poorly integrated, (b) the proportion of the elements is poor, and (c) the way the elements are put together is logical.

#### 3.1.4. PT4 Neuroticism

**Figure 4:**
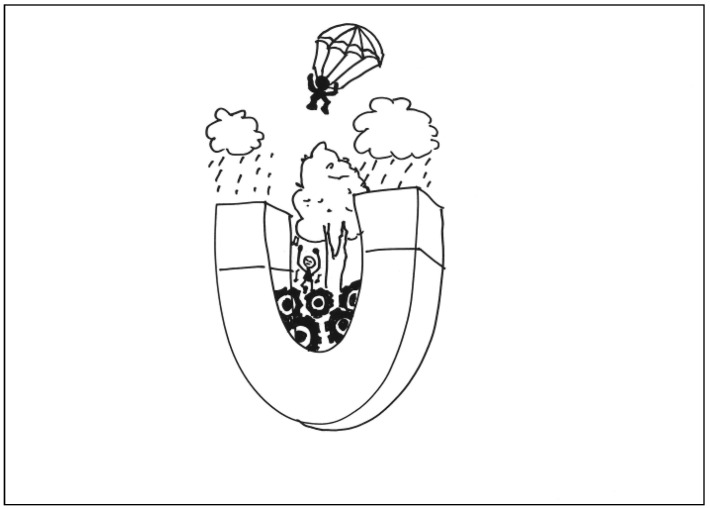
Work by PT4 Conscientiousness.

The description for TP4’s work: In a magnet kingdom, there’s a guy stuck in a gear swamp. It is raining in the forest, and a brave soldier is coming down slowly using his parachute to recuse the person stuck in the gear swamp. See Figure 4. General comments from the experts: (a) The composition is simple and precise, (b) it is highly imaginative, (c) it has a good sense of space, and (d) the composition is playful.

#### 3.1.5. PT5 Openness

**Figure 5:**
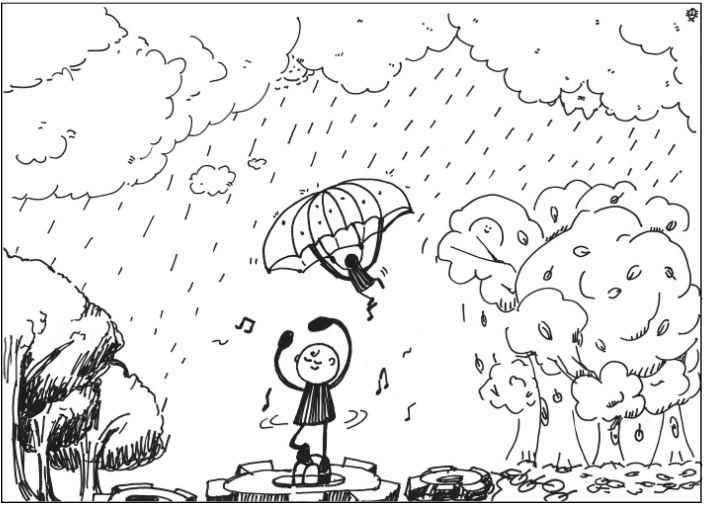
Work by PT5 Openness.

The description for TP5’s work: A parachute protects the dancing child from rain and allows the big tree to get enough rain water to grow stronger and taller. See Figure 5. General comments from the experts: (a) The composition has a good sense of space, (b) it has a good completeness, (c) it is very dynamic, and (d) the composition is well-balanced.

#### 3.1.6. APT Averaging Personality Traits

**Figure 6:**
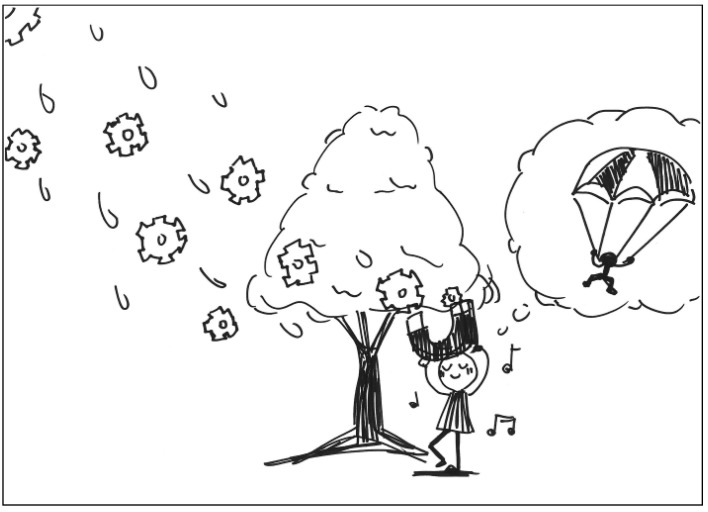
Work by APT Averaging Personality Traits.

The description for APT’s work: The weather is not good, and there is a shower of gears from the sky. Nonetheless, the robot is still passionate. The robot likes parachuting and is standing under a tree. The robot is using a huge magnet to attract all the rainwater away. The robot wants to go back to the sky to parachute again. See Figure 6. General comments from the experts: (a) The proportion of the elements is good, (b) the composition is a bit monotonous, (c) the pen stroke is coarse, and (d) the styling of the elements is too simple.

#### 3.1.7. D1 No Depression

**Figure 7:**
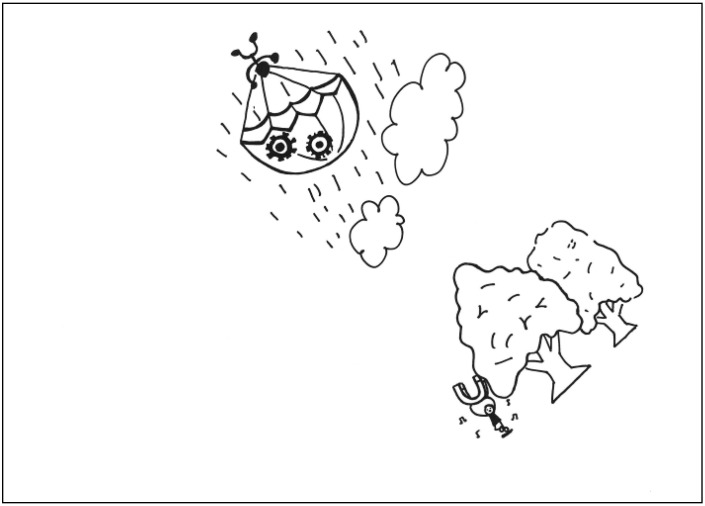
Work by D1 No Depression.

The description for D1’s work: There are two worlds on Earth; one is the happy word (the dancing person), and the other is the sorrow world (the rainy sky). People in the happy world want to use a magnet to attract those in the sorrow world to their side. The medium is the gears, while the trees symbolize the direction of the force of gravity. See Figure 7. General comments from the experts: (a) The composition fails to tell the entire story, (b) there are not enough elements and the elements are too small, and (c) the elements are not in a good proportion.

#### 3.1.8. D2 Mild Depression

**Figure 8:**
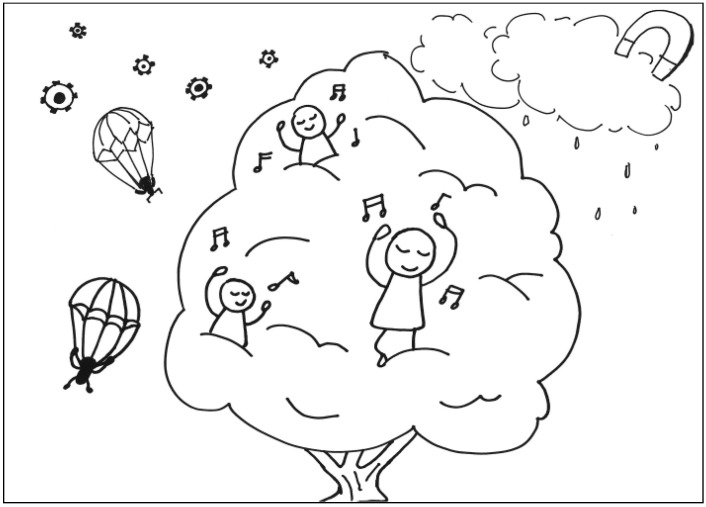
Work by D2 Mild Depression.

The description for D2’s work: We are in two different worlds: one sunny and one cloudy. The gears symbolize the Sun or stars in a fair sky. People in the sky are parachuting happily. While in the world that is raining, the magnet attracts and keeps the dark clouds away so people can dance happily in the forest. See Figure 8. General comments from the experts: (a) The image has insufficient tension, (b) the composition has a poor sense of space, and (c) there is a lack of creativity.

#### 3.1.9. D3 Moderate Depression

**Figure 9:**
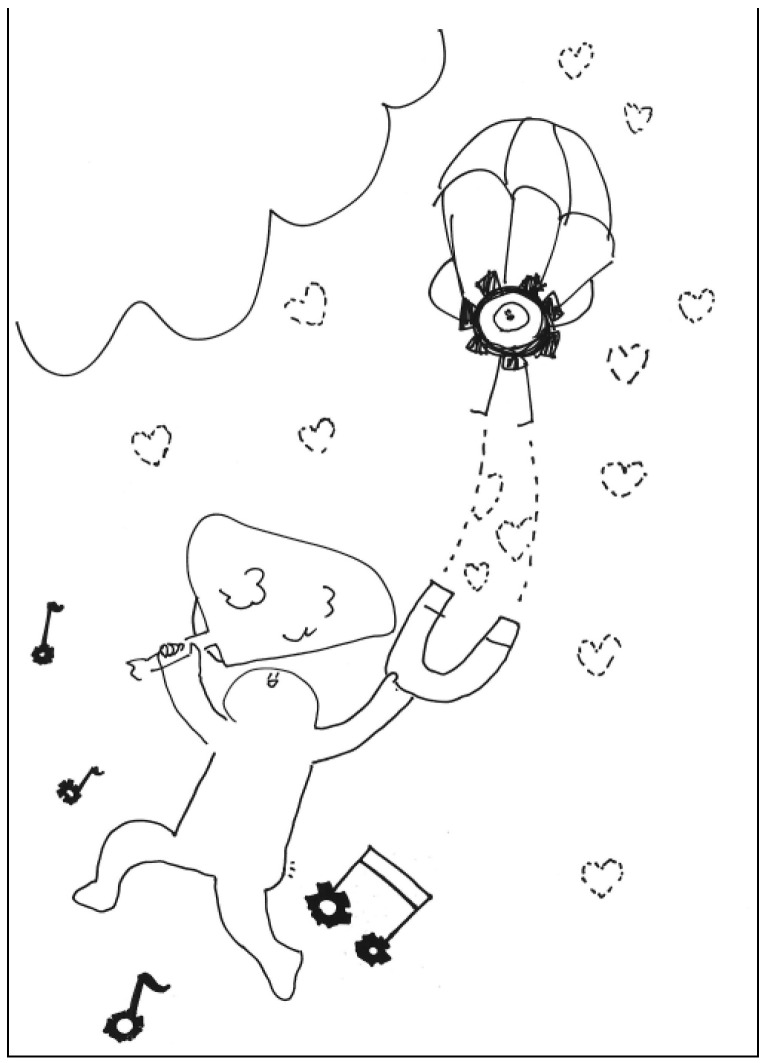
Work by D3 Moderate Depression.

The description for D3’s work: A person is doing a courtship dance in the rain of love. He is holding a tree and a magnet to attract his beloved gear person to him. He hopes that love will come down from the sky. See Figure 9. General comments from the experts: (a) The composition is too loose, (b) the pen stroke is messy, (c) there is a poor sense of space, and (d) the lines are monotonous.

#### 3.1.10. D4 Severe Depression

**Figure 10:**
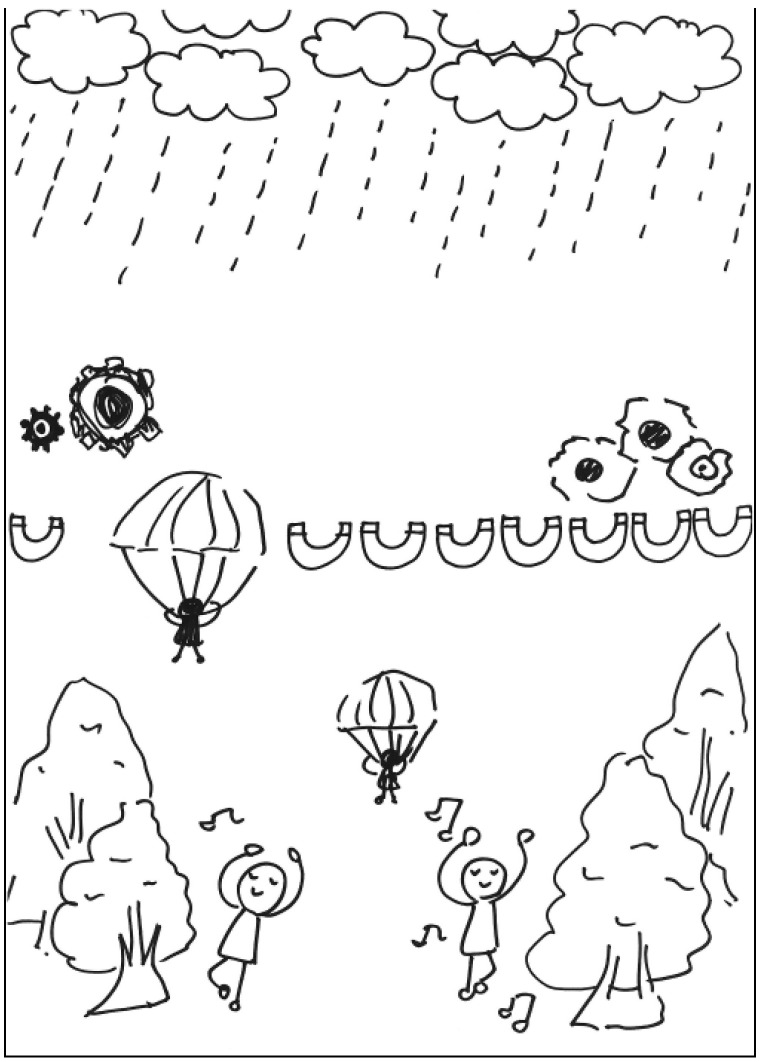
Work by D4 Severe Depression.

The description for D4’s work: People rely on nature and enjoy a happy life. Dark clouds engulfing the sky is a byproduct of industrialization and technology development. Parachuting seems dangerous, but it symbolizes people’s desire to give up technology and to return to the very beginning and live in nature. See Figure 10. General comments from the experts: (a) It has a dichotomist composition, (b) it has a theme, and (c) the composition is conservative.

#### 3.1.11. AD Averaging Depression

**Figure 11:**
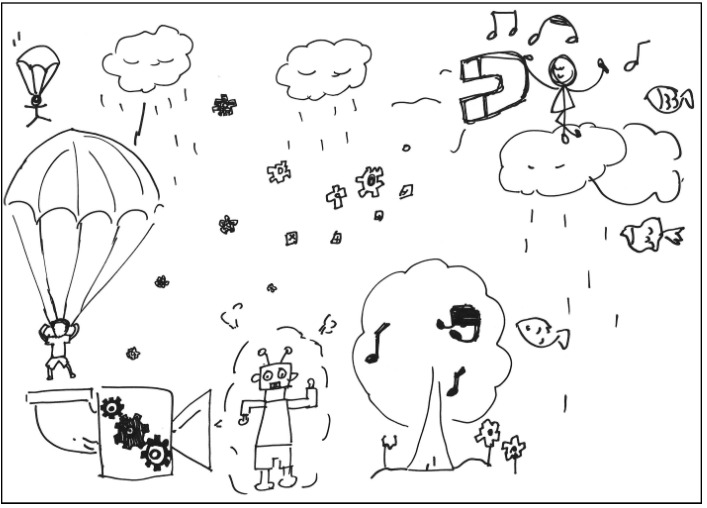
Work by AD Averaging Depression.

The description for AD’s work: Humans’ endless technology development has annoyed the god in heaven, so the god wants to take the world back. The parachuters symbolize humans’ pursuit of technology, and they are turned into robots when passing through the gear device. The god, who is standing on the clouds, is trying to recycle the world by attracting the components using a magnet. See Figure 11. General comments from the experts: (a) The composition is loose, and the elements are too scattered, (b) it has an Egyptian drawing style (a childlike style), (c) the image is presented like a flowchart, and (d) the pen stroke is messy.

### 3.2. Personality Traits and Design Performance

This study had the design performance of the personality trait groups (PT1, PT2, PT3, PT4, PT5, and APT) evaluated by five graphic design experts. The evaluation indicators were creativity (30%), aesthetics (30%), and completeness (40%), and a full score was 100 points. The scores of the personality trait groups are shown in Table 1. After completing the work in groups, each group member was asked to fill out a self-rated questionnaire. The self-rated questionnaire used a Likert scale, and the score ranges from 1 point (extremely dissatisfied) to 5 points (extremely satisfied). Table 2 shows the results from the self-rated questionnaire.

Table 1 shows that the work by PT4 Neuroticism scored the highest, and for the two indicators creativity and aesthetic, PT4 (Neuroticism) also scored the highest. This finding suggests that it is beneficial for designers with the personality trait of neuroticism to team up and collaborate. It is worth noting that PT4 Neuroticism performed well in creativity and aesthetic. Table 2 shows that PT4 Neuroticism’s self-rated score was the lowest among the six teams. For either member satisfaction or work satisfaction, the members of PT4 Neuroticism rated themselves harshly. It is possible that the team with the personality trait of neuroticism was more self-demanding, and this characteristic was reflected on the performance score of their work. In contrast, PT1 Extraversion and PT3 Conscientiousness scored the highest in the self-evaluation, but the performance scores of their works were mediocre.

### 3.3. Depression and Design Performance

This study had the design performance of the depression groups (D1, D2, D3, D4, and AD) evaluated by five graphic design experts. The evaluation indicators were creativity (30%), aesthetics (30%), and completeness (40%), and a full score was 100 points. The scores of the depression groups are shown in Table 3. After completing the work in groups, each group member was asked to fill out a self-rated questionnaire. The self-rated questionnaire used a Likert scale; the score ranges from 1 point (extremely dissatisfied) to 5 points (extremely satisfied). Table 4 shows the results from the self-rated questionnaire.

Table 3 shows that D1 No Depression performed the best on creativity, D2 Mild Depression performed the best on aesthetic, and D4 Severe Depression performed the best on completeness. Table 4 shows that D2 Mild Depression had the lowest score from the self-evaluation. For either the indicator member satisfaction or work satisfaction, the team members of D2 Mild Depression rated themselves strictly, and that is probably why D2 Mild Depression’s work scored better, especially on aesthetic.

## 4. Discussion

### 4.1. General Discussion on Personality Traits and Design Performance

Compared with the other personality trait groups, PT4 Neuroticism (M = 86), PT1 Extraversion (M = 80), and PT5 Openness (M = 77) had the best creativity from the teamwork. Guo (1994) suggested that the personality trait of neuroticism may hinder an individual’s use of creativity. However, Neuman (1999) found that the personality trait of neuroticism may have a complementary role in teamwork [41]. Therefore, even though the neuroticism personality trait may not facilitate the expression of creativity, its complementary function can be positive for teamwork.

The key qualities of the personality trait of agreeableness are friendliness, cooperativeness, altruism, and dependency. People with the personality trait of agreeableness make friends easily. They are lubrication for teamwork, but at the same time, they do not have a leadership personality. PT2 Agreeableness’s member satisfaction (M = 4.25) and work satisfaction (M = 4.5) were not great. This result may be attributed to their high dependency; everyone tries to cooperate, but when there are two different opinions, they cannot make up their minds and pick one. PT4 Neuroticism (M = 81) and PT5 Openness (M = 81) achieved the best aesthetics from the teamwork. PT1 Extraversion (M = 73) was the runner up.

In terms of member satisfaction and work satisfaction, PT5 Openness was better than PT4 Neuroticism. This finding suggests that PT5 Openness rated their teamwork performance as excellent. In addition, PT5 Openness (M = 84) and PT4 Neuroticism (M = 81) scored higher in completeness from the teamwork than other personality trait groups. If a project requires high efficiency and high completeness, people with the personality trait of openness should be on the team.

When the three indicators creativity, aesthetics, and completeness were integrated, the groups with the best design performance were PT4 Neuroticism (M = 82.5), PT5 Openness (M = 81), and PT1 Extraversion (M = 74.3). For a project that needs a good collaborative atmosphere, people with the personality trait of extraversion should be the key members of the team. If creativity is expected, the personality trait of neuroticism should be highlighted. If aesthetics is prioritized, the personality trait of openness should be preferred.

### 4.2. General Discussion on Depression and Design Performance

D1 No Depression (M = 73) and D4 Severe Depression (M = 72) had greater creativity than other depression teams. D2 Mild Depression (M = 67) and D4 Severe Depression (M = 65) achieved greater aesthetics from the teamwork than the other depression teams. D4 Severe Depression (M = 72) and D2 Mild Depression (M = 69) had greater completeness from the teamwork than other depression teams. The total scores of D4 Severe Depression (M = 70) and D2 Mild Depression (M = 69) were higher than that of the other depression teams.

According to the results of creativity, aesthetics, and completeness, and the total scores, there was no significant association between depression and design performance. The researchers here like to point out that D2 Mild Depression scored the best in design performance (their design work). However, for the two indicators member satisfaction and work satisfaction, D2 Mild Depression scored the lowest. It is possible that D2 Mild Depression was more demanding on member satisfaction and work satisfaction than other teams. A question that should be addressed in the future is whether the personality trait of self-discipline (people with depression may be more self-disciplined, for example) supports design and creativity.

About personality traits, this study found that neuroticism is associated with excellent design performance (especially in terms of the two indicators creativity and aesthetics). Guo (1994) pointed out that neuroticism is unfavorable for creativity [11], which is different from the finding of this study. In this study, several designers with the personality trait of neuroticism were grouped to create together, and working collaboratively may have reinforced or stimulated their creativity (in other words, though neuroticism is unfavorable for personal creativity, it does not affect group creativity). The effect of neuroticism on individual vs. collaborative design has to be further investigated. As for depression, the finding of this study suggests that the collaborative design performance of the team with severe depression (such as D4 Severe Depression) was not worse than of the team with mild depression (such as D2 Mild Depression). In fact, D4 Severe Depression was comprehensively rated the best. Post (1994) pointed out that those with severe depression are more creative [21], which supports this study’s viewpoint that the team with severe depression can express high creativity too.

## 5. Conclusions

This study explored the effect of creators’ personality traits and depression on the teamwork-based design performance. For personality traits, this study concludes that (1) PT4 Neuroticism performed the best in creativity and in the overall design performance from the teamwork. (2) PT1 Extraversion performed the best in creativity from the teamwork. (3) PT2 Agreeableness and PT3 Conscientiousness scored lower in creativity. People with the agreeableness personality trait prefer cooperation over conflicts, whereas those with the conscientiousness personality trait are good at offering suggestions and ideas. Because they both lack a leadership personality trait, the result of their teamwork was suboptimal. (4) In terms of the completeness of the design works, PT5 Openness performed the best. For a group that requires efficiency and needs to complete a project rapidly in a limited amount of time, people with the openness personality trait are preferred.

Overall, PT4 Neuroticism, PT5 Openness, and PT1 Extraversion did the best in the group performance. It was observed that when members of PT1 Extraversion were in discussion, the atmosphere was positive and spirited. Therefore, companies and organizations can make use of this personality trait to improve team performance.

As for depression, this study made the following conclusions: (1) D4 Severe Depression’s overall rating was better than teams with a lower level of depression. The quality of design of people with severe depression when working in groups was good. (2) In terms of teamwork, there was no significant association between the severity of depression and design performance (i.e., creativity, aesthetics, and completeness). However, people with severe depression were indeed most creative, which confirms the theories of other researchers. (3) It was observed that the teamwork atmosphere of D3 Moderate Depression and D4 Severe Depression (M = 72) was heavy and negative. Even though severe depression may lead to good design performance, it makes teamwork not so pleasant. A solution to the problem above is to have people with the openness personality trait on the team.

Teamwork (including cross-disciplinary collaboration) will be the mainstream in the future. Personality traits have been extensively explored, and it has been shown that team performance can be substantially enhanced if people on a team are equipped with good communication skills and professional capabilities. Personality traits and the level of depression can affect the overall performance of teamwork. In fact, each personality trait or emotional expression has its unique way to enhance team performance. For example, some may enhance the creativity part of the performance, while others may enhance the aesthetic component of the performance. Therefore, the manager at each project stage should have people of different personality traits added to or removed from the team in order to maximize teamwork through personality traits and emotional expression.

This study explored the effect of creators’ personality traits and depression on teamwork-based design performance. Tony Wagner, a co-director of the Change Leadership Group at the Harvard Graduate School of Education, suggested the four critical skills that should be possessed by future workforces are critical thinking, communication, collaboration, and creative problem solving [42]. Teamwork skills will be a core value. This study helps the decision-maker of an organization grasp the personality traits and emotional expression for building group cohesion. It also enables creators to better understand and combine different personality traits and depression levels to optimize teamwork-based design performance.

## Figures and Tables

**Table 1:** Scores of Personality Trait Groups’ Works.

Group	PT1	PT2	PT3	PT4	PT5	APT	Average
Creativity	80	61	66	86	77	71	74
Ranking	2	6	5	1	3	4	
Aesthetic	73	60	58	81	81	67	70
Ranking	3	5	6	1	1	4	
Completeness	71	63	63	81	84	71	72
Ranking	3	5	5	2	1	3	
Total	74	62	63	83	81	70	72
Ranking	3	6	5	1	2	4	

**Table 2:** Personality Trait Groups’ Self-Rated Performance.

Group	PT1	PT2	PT3	PT4	PT5	APT	Average
Member satisfaction	5.00	4.25	5.00	4.00	4.75	4.50	4.58
Ranking	1	5	1	6	3	4	
Work satisfaction	5.00	4.50	5.00	4.00	4.25	4.50	4.54
Ranking	1	3	1	6	5	3	

**Table 3:** Scores of Depression Groups’ Works.

Group	D1	D2	D3	D4	AD	Average
Creativity	73	70	69	72	65	70
Ranking	1	3	4	2	5	
Aesthetic	63	67	63	65	59	63
Ranking	3	1	3	2	5	
Completeness	66	69	66	72	67	68
Ranking	4	2	4	1	3	
Total	67	69	66	70	66	68
Ranking	3	2	4	1	4	

**Table 4:** Depression Groups’ Self-Rated Performance.

Group	D1	D2	D3	D4	AD	Average
Member satisfaction	4.75	4.00	4.75	4.75	4.75	4.60
Ranking	1	5	1	1	1	
Work satisfaction	4.50	4.00	4.50	4.75	4.75	4.50
Ranking	1	5	1	1	1	

## Data Availability

Data can be requested from the corresponding author.
